# Oncologic outcome and safety profile of first-line enfortumab vedotin plus pembrolizumab vs. conventional chemotherapy in advanced urothelial cancer

**DOI:** 10.37349/etat.2026.1002379

**Published:** 2026-07-06

**Authors:** Makito Miyake, Nobutaka Nishimura, Yuki Oda, Takuto Shimizu, Takeshi Inoue, Satoshi Anai, Keichi Sakamoto, Yoshiaki Matsumura, Yuta Toyoshima, Daiki Ichii, Kota Iida, Takashi Iwamoto, Norimi Takamatsu, Atsushi Tomioka, Hiroaki Yamamoto, Fumisato Maesaka, Kiyohide Fujimoto, on behalf of the Nara Urological Research Treatment Group

**Affiliations:** IRCCS Istituto Romagnolo per lo Studio dei Tumori (IRST) “Dino Amadori”, Italy; ^1^Department of Urology, Nara Medical University, Kashihara 634-8521, Japan; ^2^Department of Urology, Nara Prefecture General Medical Center, Nara 630-8581, Japan; ^3^Department of Urology, Nara Prefecture Seiwa Medical Center, Ikoma 636-0802, Japan; ^4^Department of Urology, Nara City Hospital, Nara 630-8305, Japan; ^5^Department of Urology, Osaka Kaisei Hospital, Osaka 532-0003, Japan.; ^6^Department of Urology, Yamatotakada Municipal Hospital, Yamatotakada 635-8501, Japan.; ^7^Department of Urology, Tane General Hospital, Osaka 550-0025, Japan; ^8^Department of Urology, Osaka Gyoumeikai Hospital, Osaka 554-0012, Japan; ^9^Department of Urology, Matsusaka Chuo General Hospital, Matsusaka 515-0818, Japan; ^10^Department of Urology, Saiseikai Chuwa Hospital, Sakurai 633-0054, Japan.; ^11^Department of Urology, Minami Nara General Medical Center, Yoshino 638-0833, Japan.; ^12^Department of Urology, Koseikai Takai Hospital, Tenri 632-0006, Japan.

**Keywords:** urothelial carcinoma, chemotherapy, response, outcome, enfortumab vedotin, pembrolizumab, adverse event

## Abstract

**Aim:**

The aim of this multicenter study was to assess patient characteristics and short-term survival outcomes of first-line (1L) enfortumab vedotin plus pembrolizumab (EVP) as compared with conventional chemotherapy in locally advanced or metastatic urothelial carcinoma (la/mUC).

**Methods:**

The database included 642 patients with la/mUC diagnosed between January 2008 and December 2025 at 12 collaborating hospitals. Baseline characteristics and follow-up data, including overall and organ-specific objective tumor response according to the RECIST v1.1, progression-free survival, and duration of response, were compared among the 1L regimens. Treatment-related adverse events (TRAEs) were graded according to the CTCAE v5.0 in patients treated with 1L EVP.

**Results:**

The objective response and disease control rate were higher with 1L EVP than with chemotherapy (66% vs. 42% and 83% vs. 68%, respectively). The organ-specific response rate for liver metastatic lesions was 85%. Median progression-free survival (95% confidence interval) for 1L EVP, gemcitabine plus cisplatin, and gemcitabine plus carboplatin was 14.5 months (10.5–not determined), 10.5 months (8.4–13.3), and 9.2 months (6.7–14.2), respectively. In the safety analysis set including 40 patients, all-grade TRAEs occurred in 36 (90%) patients, including grade 1–2 events in 30 (75%) and grade 3–4 toxicities in 6 (15%). Grade 3–4 TRAEs included skin toxicity (7.5%), anorexia (5.0%), anemia (5.0%), gastrointestinal disorders (2.5%), renal dysfunction (2.5%), and interstitial lung disease (2.5%). The median number of administered EV cycles was 4 (range, 1–13), 5 (2–13), and 10 (4–13) in the overall, responder, and complete-response populations, respectively. EV dose modifications and interruptions were frequent in the initial 2 months but rare thereafter.

**Conclusions:**

This multicenter study provides real-world evidence on short-term outcomes and safety with 1L EVP, highlighting its impact on the evolving treatment landscape for la/mUC.

## Introduction

Carcinoma arising from the urinary tract mucosa (renal pelvis, ureter, bladder, and urethra) is the sixth most common malignancy in the United States [1], with urothelial carcinoma (UC) being the predominant histologic subtype. According to Global Cancer Statistics 2020, bladder cancer is the seventh most common cancer among men worldwide and the 13th most common cancer in Japan [2, 3]. Locally advanced or metastatic UC (la/mUC) is an aggressive and lethal disease associated with a poor prognosis. The treatment landscape for la/mUC has evolved substantially with the introduction of immune checkpoint inhibitors (ICIs) and antibody-drug conjugates (ADCs) [4]. Based on the positive outcomes of the EV-302/KN-A39 trial (NCT04223856) [5], first-line (1L) enfortumab vedotin plus pembrolizumab (EVP) combination therapy was approved by the U.S. Food and Drug Administration in December 2023, the European Medicines Agency (EMA) in August 2024, and the Pharmaceuticals and Medical Devices Agency (PMDA) in Japan in September 2024. Updated overall survival (OS) results from EV-302/KN-A39 after a median follow-up of approximately 2.5 years showed a median OS of 33.8 months among patients in the EVP arm [6]. Currently, this combination therapy is the new standard of care recommended in several guidelines [7–10].

An exploratory analysis of responders (complete response [CR] or partial response [PR] as the best objective response) from the EV-302/KN-A39 trial, presented at the ASCO 2025 Annual Meeting, reported a durable response, with 74% and 23% probabilities of no progression in the CR and PR populations, respectively [11]. In another subgroup analysis, EVP continued to demonstrate superior oncologic efficacy compared with chemotherapy (CT) across prespecified subgroups, such as lymph node (LN) metastasis (mets) only, visceral mets, absence of liver mets, and presence of liver mets, with the magnitude of benefit consistent with the overall population [12]. Recently, Kardoust Parizi et al. [13] conducted a systematic review and network meta-analysis to evaluate the objective response rates (ORRs) to CT, ICIs, and ADCs across different metastatic sites. However, evidence regarding organotropism-related differences in treatment response to EVP remains limited. Recently, several studies have reported evidence regarding the epidemiology, histological features, diagnostic approaches, and novel molecular findings in UC [14–17].

It has been over 1 year since the approval of 1L EVP for la/mUC in Japan; however, real-world data on this new treatment remain limited. The present study reports a retrospective analysis from an EVP-treated patient registry, aiming to evaluate patient characteristics, treatment patterns, best objective response to 1L EVP, and the safety of EVP compared with conventional 1L CT in a real-world setting.

## Materials and methods

### Data collection

This retrospective, multi-institutional study was approved by the Nara Medical University Ethics Committee (protocol ID: 2891) and conducted in accordance with the principles of the Declaration of Helsinki. Informed consent was obtained from participants through posters and/or the institutional website using an opt-out method [18]. We retrospectively reviewed electronic medical records from 642 patients with la/mUC diagnosed between January 2008 and December 2025 at 12 collaborating hospitals. Baseline characteristics, including age, sex, smoking history, Eastern Cooperative Oncology Group Performance Status (ECOG-PS) score, Charlson Comorbidity Index (CCI), primary tumor site, radical surgery status, laboratory data, and metastatic and target lesions, were collected. Follow-up data, including tumor response and duration of response (DOR) based on radiographic examinations, were assessed from the start of 1L therapy to the last documented follow-up or data lock (March 2026).

Patients with treatment-naïve la/mUC were evaluated using the Enfortumab Vedotin Ineligible Criteria (EVITA), defined as follows: i) Hemoglobin A1c ≥ 8% (if this parameter was unavailable, it was replaced by baeline glucose > 150 mg/dL in two consecutive blood samples taken 1 week apart); ii) Grade ≥ 2 sensory or motor neuropathy; iii) Any corneal or retinal abnormality; iv) Estimated glomerular filtration rate (eGFR) < 45 mL/min/1.73 m^2^; v) ECOG-PS score ≥ 2.

In this study, an estimated GFR < 45 mL/min/1.73 m^2^, calculated using the Japanese equation for estimated GFR from serum creatinine, was adopted as a substitute for the original criterion “creatinine clearance or GFR < 45 mL/min” for practical purposes [19–21].

### Regimens of the 1L systemic treatment

Because of the retrospective nature of this study, the regimen, dose reduction, dose interruption, treatment duration, and number of cycles were determined by the attending physicians in consultation with the patients, based on factors such as adverse events, organ function, CCI, and ECOG-PS score. Intravenous EV was administered on days 1 and 8, and intravenous pembrolizumab at a dose of 200 mg (or 400 mg every 6 weeks) was given after the enfortumab vedotin infusion on day 1 of each 3-week cycle. For EV administration, a dose of 1.25 mg/kg was considered the standard induction regimen 5. Dose modifications for EV were implemented in stepwise 0.25 mg/kg reductions as follows: 1.25, 1.0, 0.75, and 0.5 mg/kg as the minimal dose. Interruptions of EV and/or pembrolizumab were carefully considered, primarily based on adverse events. Conventional platinum-based CT (Pl-CT) regimens—including gemcitabine plus cisplatin (GC), gemcitabine plus carboplatin (GCarbo), and dose-dense methotrexate, vinblastine, doxorubicin, and cisplatin (DD-MVAC)—were administered as previously reported [22, 23]. The analysis also included patients receiving 1L non-Pl-CT, such as taxane-based regimens, which were classified as “others.”

### Assessment for tumor response

The overall objective response was categorized as CR, PR, stable disease (SD), or progressive disease (PD) according to the Response Evaluation Criteria in Solid Tumors (RECIST) version 1.1 [24], based on computed tomography and/or magnetic resonance imaging, as previously described. In the analysis of 1L EVP therapy, the percentage change from baseline in the sum of the diameters of measurable target lesions at the time of the objective response for each patient was illustrated using waterfall plots, and longitudinal changes in these diameters for each patient were depicted using spider plots.

Organotropism-related differences in treatment response (organ-specific response) were evaluated separately for primary or locally recurrent lesions, LN mets, lung mets, and liver mets. In patients with metastases involving multiple sites or organs, only the responses of the lesions of interest were considered to assess organotropism-related differences in treatment response. The response (CR, PR, SD, or PD) was determined in the same manner as the overall objective response.

This study investigated the prognostic impact of several blood-based inflammation and nutrition markers in la/mUC patients treated with 1L EVP, including the neutrophil-to-lymphocyte ratio (NLR), monocyte-to-lymphocyte ratio (MLR), platelet-to-lymphocyte ratio (PLR), systemic inflammation response index (SIRI, defined as monocyte count × neutrophil count/lymphocyte count), systemic immune-inflammation index (SII, defined as platelet count × neutrophil count/lymphocyte count), and C-reactive protein (CRP)–albumin–lymphocyte (CALLY) index (calculated as serum albumin × total lymphocyte count/[CRP × 10^4^]) [25].

### Assessment of treatment-related adverse events (TRAEs) in the 1L EVP group

TRAEs were graded according to the National Cancer Institute Common Terminology Criteria for Adverse Events (CTCAE) version 5.0, based on clinical laboratory tests, physical examinations, and medical interviews conducted by physicians or investigators. The dates of AE onset and recovery were monitored. Adverse Events of Special Interest (AESI) identified for this study included five categories: skin toxicity, peripheral neuropathy, dysgeusia, thyroid disorder, and interstitial lung disease (ILD). Events that changed in grade for individual patients were analyzed independently using Kaplan-Meier methods for each respective grade. Patients without a corresponding AE of a particular grade were censored in the time-to-event analysis for that grade at the earlier of the following: last dose date plus 1 month, data cutoff, or end-of-study date.

### Statistical analysis

Statistical analyses were performed, and figures were generated using GraphPad Prism version 10 (GraphPad Software, San Diego, CA, USA). Statistical significance was set at *p* < 0.05. Clinicopathological characteristics were compared using the chi-squared test, Fisher’s exact test, Mann-Whitney *U* test, and Kruskal-Wallis test, as appropriate. Disease progression was defined as radiographic PD according to RECIST version 1.1 or death from any cause. Progression-free survival (PFS) was estimated from the start of 1L therapy using the Kaplan-Meier method and compared between groups using the log-rank test, with *p* values provided.

## Results

### Comparison between the 1L EVP and non-EVP groups diagnosed after the approval of 1L EVP use

The use of 1L EVP for la/mUC was approved by the PMDA of Japan on September 27, 2024. A total of 65 patients with systemic therapy-naïve la/mUC were diagnosed between October 2024 and December 2025. Of these, 55 (85%) received 1L EVP therapy, while 10 (15%) received non-EVP therapy. Among the 10 patients in the non-EVP group, five received GCarbo, three received DD-MVACarbo [26], one received GC, and one did not receive any systemic therapy (best supportive care [BSC]). Table S1 lists details of patients diagnosed with la/mUC between October 2024 and December 2025 who did not receive 1L EVP therapy, including reasons for not selecting EVP. Baseline characteristics of the patients are compared between the two groups (Table 1), showing that younger age (74 vs. 79 years), absence of diabetes mellitus (13.0% vs. 40.0%), presence of liver mets 24.0% vs. 0.0%), larger tumor volume (median sum, 49 mm vs. 32 mm), better PS (ECOG-PS ≤ 1: 89% vs. 50%), and a lower number of EVITA were significantly or marginally associated with the selection of EVP.

**Table 1:** Baseline characteristics of patients with la/mUC diagnosed between October 2024 and December 2025: comparison between 1L EVP vs 1L non-EVP cases.

**Variables**		**1L EVP**	**Non-EVP**	***P* value**
*n*		55	10	-
Age, years-old	Median (IQR)	74 (66–78)	79 (77–85)	0.01
Sex	Male	40 (72.7%)	8 (80.0%)	0.72
	Female	15 (27.3%)	2 (20.0%)	
Smoking history	Never	17 (30.9%)	3 (30.0%)	0.97
	Former	20 (36.4%)	4 (40.0%)	
	Current	10 (18.2%)	2 (20.0%)	
	Unknown	8 (14.5%)	1 (10.0%)	
Charlson Comorbidity Score-Category	None (score 0)	29 (52.7%)	6 (60.0%)	0.78
	Mild (score 1 or 2)	22 (40.0%)	4 (40.0%)	
	Moderate (score 3 or 4)	4 (7.3%)	0	
	Severe (score 5 or more)	0	0	
Radical surgery	None	30 (54.5%)	6 (60.0%)	0.38
	Cystectomy	16 (29.1%)	1 (10.0%)	
	Nephroureterectomy	8 (14.5%)	3 (30.0%)	
	Both	1 (1.8%)	0	
Diabetes mellitus	No	48 (87.3%)	6 (60.0%)	0.09
	Yes	7 (12.7%)	4 (40.0%)	
Clinical T category, the 8th edition	Tis	3 (5.5%)	0	0.55
	T1	6 (10.9%)	1 (10.0%)	
	T2	10 (18.2%)	2 (20.0%)	
	T3	16 (29.1%)	5 (50.0%)	
	T4	20 (36.4%)	2 (20.0%)	
Primary disease	Bladder cancer	29 (52.7%)	6 (60.0%)	0.75
	UTUC	26 (47.3%)	4 (40.0%)	
Unresectable or metastatic lesions	Local lesion	31 (56.4%)	5 (50.0%)	0.76
	Lymph nodes	40 (72.7%)	7 (70.0%)	0.73
	Lung	17 (30.9%)	3 (30.0%)	1.00
	Liver	13 (23.6%)	0	0.68
	Bone	9 (16.4%)	0	0.20
	Peritoneum	2 (3.6%)	0	1.00
	Retroperitoneum	7 (12.7%)	0	0.34
	Adrenal grand	1 (1.8%)	0	1.00
SLD at baseline (mm), RECIST v1.1	Median (IQR)	49 (34–81)	32 (25–34)	0.04
EVITA	HbA1c ≥ 8%	1 (1.8%)	1 (10.0%)	0.28
	Grade ≥ 2 sensory or motor neuropathy	0	0	NA
	Any corneal or retinal abnormality	1 (1.8%)	0	NA
	eGFR < 45 mL/min/1.73 m^2^	22 (40.0%)	6 (60.0%)	0.75
	ECOG-PS ≥ 2	6 (10.9%)	5 (50.0%)	<0.01
Number of EVITA	0	29 (52.7%)	1 (10.0%)	<0.01
	1	22 (40.0%)	6 (60.0%)	
	2	4 (7.3%)	3 (30.0%)	
Laboratory data, median (IQR)	Neutrophil, ×10^3^/µL	4.1 (3.1–5.6)	5.3 (4.3–6.5)	0.33
	Albumin, g/dL	3.8 (3.4–4.1)	3.8 (3.3–4.0)	0.68
	eGFR, mL/min/1.73 m^2^	49.0 (39.5–61.2)	40.8 (35.6–51.4)	0.34
	ACTH, pg/mL	26.4 (18.6–37.0)	21.7 (19.9–26.0)	0.22
	Cortisol, μg/dL	12.3 (8.5–17.01)	9.6 (8.7–11.4)	0.32
	FT3, ng/dL	2.50 (2.12–2.72)	2.7 (2.6–2.7)	0.20
	FT4, ng/dL	1.11 (0.99–1.28)	0.9 (0.9–1.0)	0.18
	TSH, μIU/mL	1.94 (1.34–3.06)	1.9 (1.7–3.8)	0.78

1L: first-line; ACTH: adrenocorticotropic hormone; ECOG-PS: Eastern Cooperative Oncology Group Performance Status; eGFR: estimated glomerular filtration rate; EVITA: Enfortumab Vedotin Ineligible Criteria; EVP: enfortumab vedotin plus pembrolizumab; FT3: free triiodothyronine; FT4: free thyroxine; IQR: interquartile range; la/mUC: locally advanced or metastatic urothelial carcinoma; NA: not available; RECIST: Response Evaluation Criteria in Solid Tumors; SLD: sum of longest diameter; TSH: thyroid-stimulating hormone; UTUC: upper urinary tract urothelial carcinoma.

### Disease characteristics of the overall population

A multicenter database included 642 patients with mUC diagnosed between January 2008 and December 2025; among these, 65 (10.1%) received BSC without any systemic therapy, whereas 55 (8.6%), 281 (43.8%), 13 (2.0%), 178 (27.7%), and 50 (7.8%) patients were treated with 1L EVP, GC, DD-MVAC, GCarbo, and other regimens, respectively. Baseline characteristics of the six groups are summarized in Table S2, demonstrating significant differences in several clinical characteristics. Notably, renal function, as assessed by eGFR, was higher in the GC and DD-MVAC groups (eGFR ≥ 45 mL/min/1.73 m^2^) compared with the EVP, GCarbo, and other groups (eGFR < 45 mL/min/1.73 m^2^).

### Tumor response analysis of patients with la/mUC treated with 1L systemic therapy

**Figure 1:**
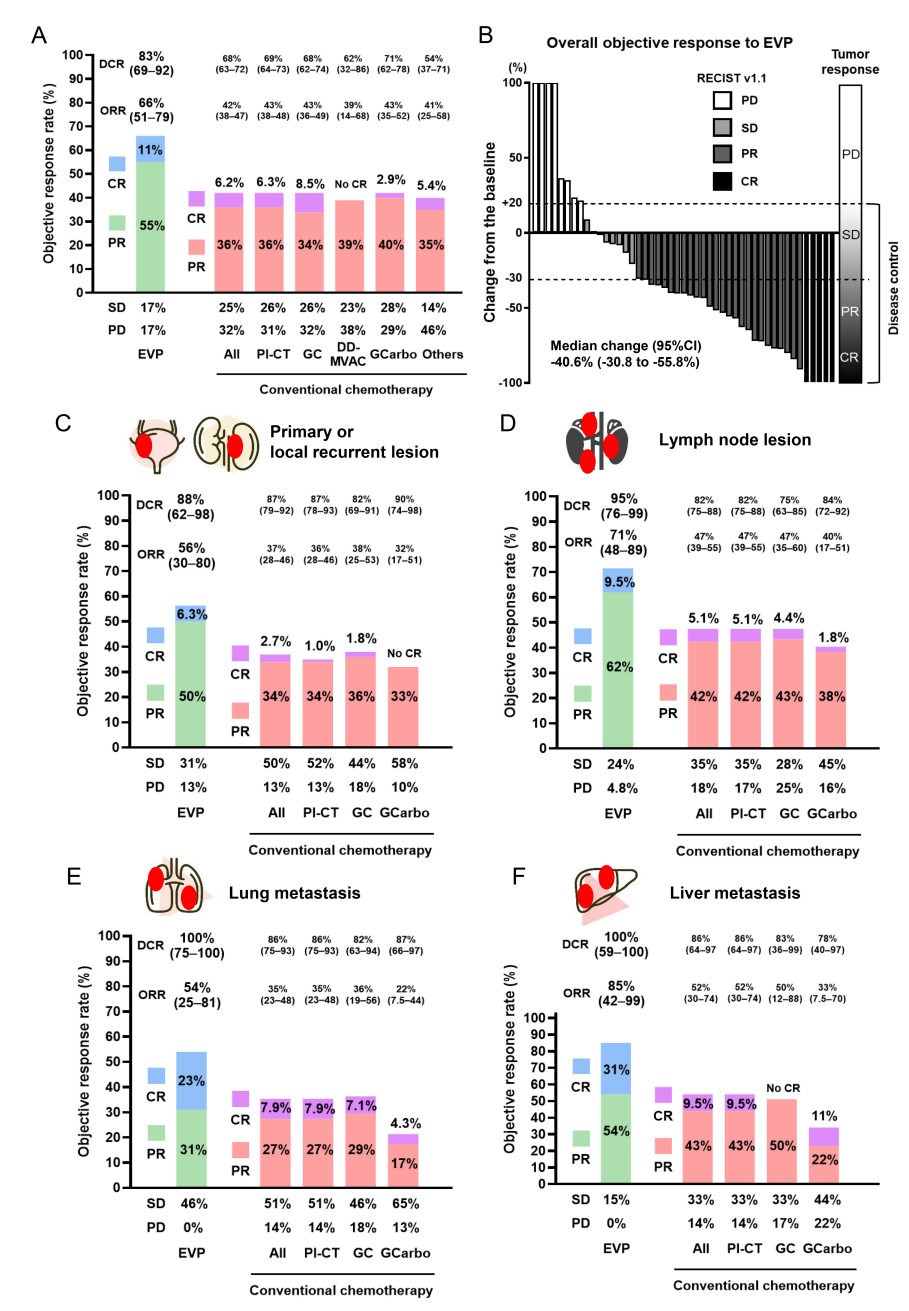
**Overall objective response and organotropism-related differential treatment response to 1L systemic therapy in la/mUC.** (**A**) ORR compared between 1L EVP and conventional chemotherapy. (**B**) Waterfall plot showing the percentage change in the sum of target lesion diameters from baseline at the best objective response for each patient treated with 1L EVP. Organotropism-related differential treatment responses to 1L EVP and conventional chemotherapy for (**C**) primary or locally recurrent lesions, (**D**) lymph node mets, (**E**) lung mets, and (**F**) liver mets. 1L: first-line; CR: complete response; DD-MVAC: dose-dense methotrexate, vinblastine, adriamycin, and cisplatin; EVP: enfortumab vedotin plus pembrolizumab; GC: gemcitabine plus cisplatin; GCarbo, gemcitabine plus carboplatin; la/mUC: locally advanced or metastatic urothelial carcinoma; ORR: objective response rate; PD: progressive disease; Pl-CT: platinum-based chemotherapy; PR: partial response; SD: stable disease.

The ORR, disease control rate (DCR), and PD rate for 1L EVP therapy were 66% (CR: 11% and PR: 55%), 83% (SD: 17%), and 17%, respectively, whereas those for conventional CT were 42% (CR: 6.2% and PR: 36%), 68% (SD: 25%), and 32%, respectively (Figure 1A). The change in tumor size from baseline was illustrated in waterfall plots, showing a median change of –40.6% (95% confidence interval [CI]: –30.8% to –55.8%) (Figure 1B). Although there was no substantial difference in tumor response among conventional CT regimens (ORRs: 39–43%), the PD rate in the “other” group was lower compared with Pl-CT (32% *vs* 46%). Organotropism-related differences in treatment response were evaluated separately for primary or locally recurrent lesions, LN mets, lung mets, and liver mets (Figures 1C–F). The corresponding ORRs were 56%, 71%, 54%, and 85%, respectively. Notably, the CR, PR, and DCR rates for liver metastases were 31%, 54%, and 100%, respectively, indicating that organotropism for 1L EVP was highest in liver metastatic lesions.

**Figure 2:**
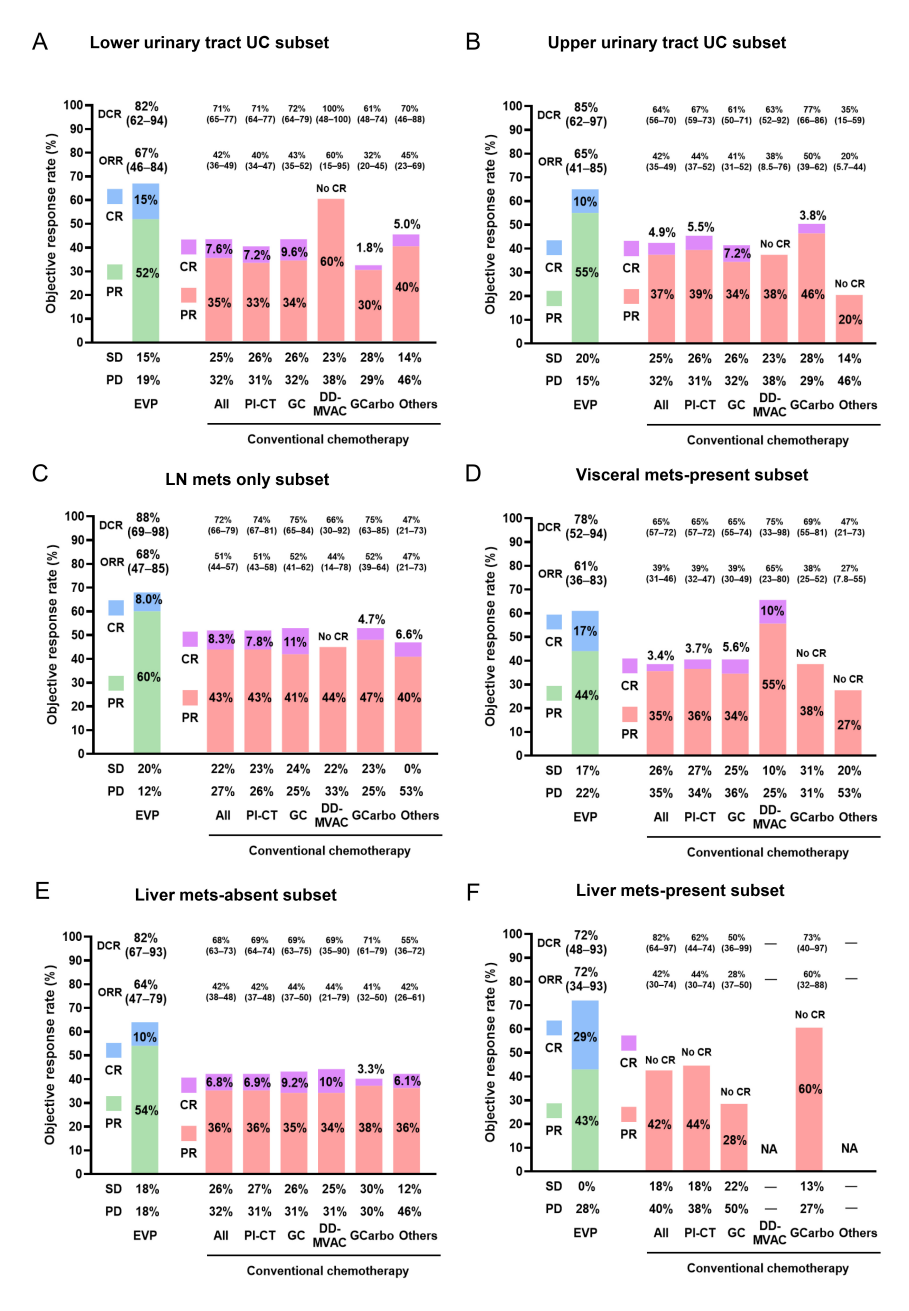
**Overall objective response to 1L systemic therapy across different disease subsets in la/mUC.** ORR compared between 1L EVP and conventional chemotherapy across the following disease subsets: (**A**) lower urinary tract urothelial carcinoma; (**B**) upper urinary tract urothelial carcinoma; (**C**) LN mets only subset; (**D**) visceral mets-present subset; (**E**) liver mets-absent subset; and (**F**) liver mets–present subset. 1L: first-line; CR: complete response; DD-MVAC: dose-dense methotrexate, vinblastine, adriamycin, and cisplatin; EVP: enfortumab vedotin plus pembrolizumab; GC: gemcitabine plus cisplatin; GCarbo, gemcitabine plus carboplatin; la/mUC: locally advanced or metastatic urothelial carcinoma; ORR: objective response rate; PD: progressive disease; Pl-CT: platinum-based chemotherapy; PR: partial response; SD: stable disease.

Next, overall ORRs were compared among 1L systemic therapies across different disease subsets, including the lower urinary tract UC subset, upper urinary tract UC subset, LN mets only subset, visceral mets-present subset, liver mets-absent subset, and liver mets-present subset (Figure 2). Overall, 1L EVP demonstrated sustained oncologic efficacy compared with conventional CT across all disease subsets. Notably, the response to 1L EVP was relatively higher in the liver mets-present subset compared with other subsets (ORR: 72% vs. 61–68%). The response to 1L EVP in the upper urinary tract UC subset was comparable with the lower urinary tract UC subset (ORR: 65% vs. 67%). The comparison of clinical background between the bladder UC and the upper urinary tract UC subsets demonstrated lower eGFR in the latter subset (Table S3).

### DOR in responders who achieved CR or PR in 1L therapy

**Figure 3:**
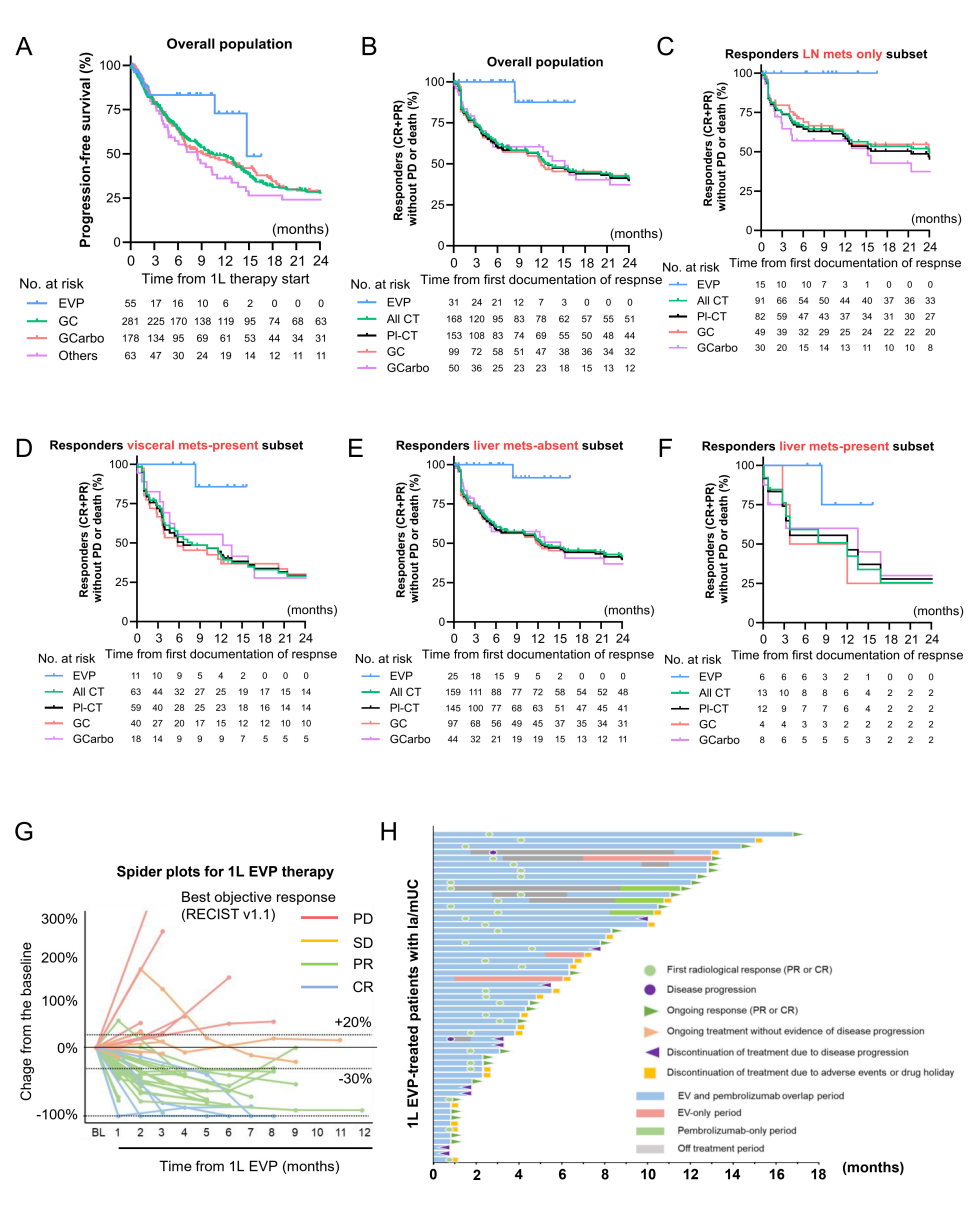
**Progression-free survival and duration of response to 1L systemic therapy in la/mUC.** (**A**) PFS curves comparing 1L EVP with conventional chemotherapy in the overall population. DOR compared between 1L EVP and conventional chemotherapy across different disease subsets: (**B**) overall population; (**C**) LN mets only subset; (**D**) visceral mets–present subset; (**E**) liver mets-absent subset; and (**F**) liver mets-present subset. Only two patients who responded to 1L EVP experienced subsequent disease progression. (**G**) Spider plot demonstrating tumor growth or shrinkage from baseline over time. Lines are color-coded according to objective response to EV therapy. Horizontal dashed lines represent partial response (≥ 30% decrease) and progressive disease (≥ 20% increase). (**H**) Swimmer plot of 55 patients receiving 1L EVP illustrating the time to first radiologic response, treatment discontinuation of EV and pembrolizumab, disease course, and DOR in responders. Legends are shown within the figure. 1L: first-line; CR: complete response; DD-MVAC: dose-dense methotrexate, vinblastine, adriamycin, and cisplatin; EVP: enfortumab vedotin plus pembrolizumab; GC: gemcitabine plus cisplatin; GCarbo, gemcitabine plus carboplatin; la/mUC: locally advanced or metastatic urothelial carcinoma; ORR: objective response rate; PD: progressive disease; PFS: progression-free survival; Pl-CT: platinum-based chemotherapy; PR: partial response; SD: stable disease.

Figure 3A presents PFS from the start of 1L systemic therapy in the overall population across selected 1L regimens. The median PFS (95%CI) for EVP, GC, GCarbo, and other regimens were 14.5 months (10.5–not determined), 10.5 months (8.4–13.3), 9.2 months (6.7–14.2), and 8.5 months (4.7–11.0), respectively. No significant difference was observed between 1L EVP and other CT groups, partly due to the limited follow-up duration of the EVP group (log-rank *p* = 0.14). DOR among responders to 1L therapy was compared using Kaplan-Meier estimation across the 1L regimens, showing a consistent durable response across different disease subsets (Figures 3B–F). The majority of patients who achieved an objective response to 1L EVP therapy demonstrated a durable response. A spider plot was generated to visualize longitudinal changes in tumor size among patients receiving 1L EVP (Figure 3G). A swimmer plot of patients receiving 1L EVP illustrated the time to first radiologic response, treatment discontinuation of EV and pembrolizumab, disease course, and DOR among patients achieving CR or PR (Figure 3H). In la/mUC patients treated with 1L EVP, there was no association of PFS and OS with several blood-based inflammation and nutrition markers including CRP, NLR, MLR, PLR, SIRI, SII, PNI, and CALLY index (Figure S1). Among 55 patients treated with 1L EVP, 5 (9.1%) received subsequent systemic therapy consisting of GC in two, GCarbo in one, DD-MVAC in one, and FGFR3 inhibitors in one patient.

### Assessment of safety profile and dose reduction/dose interruption of EV and pembrolizumab

**Figure 4:**
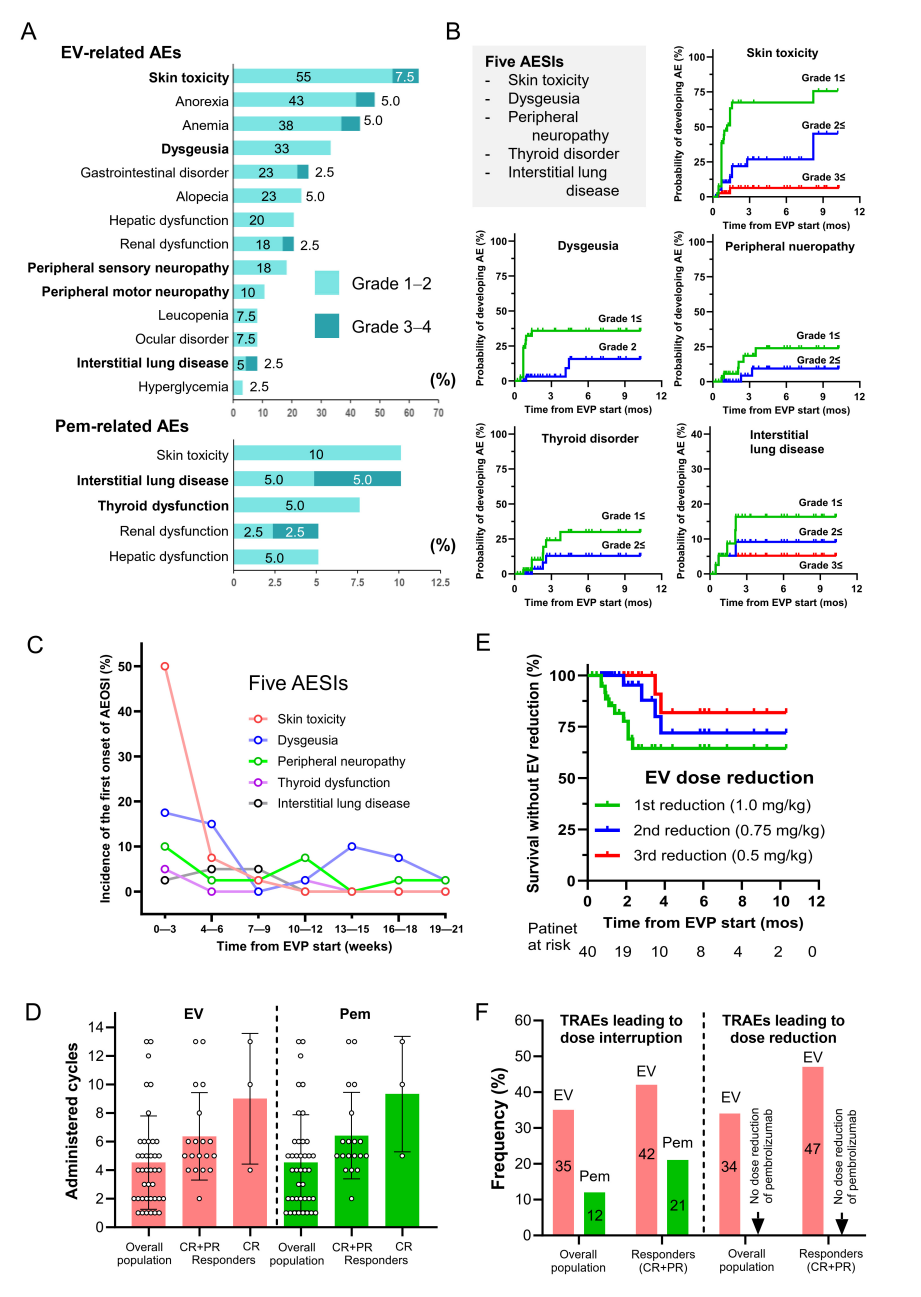
**Safety profile and dose reduction or interruption of 1L EVP in la/mUC.** (**A**) EV-related and pembrolizumab-related adverse events (AEs) were evaluated separately based on the discretion of physicians or investigators. (**B**) AESI identified in this study included five AEs: skin toxicity, peripheral neuropathy, dysgeusia, thyroid disorder, and ILD. Events that changed in grade per patient were reported independently in Kaplan-Meier analyses for each respective grade. Patients without a corresponding AE of a particular grade were censored in the time-to-event analysis at the last dose date plus 1 month, data cutoff, or end-of-study date, whichever occurred first. (**C**) Incidence of first AESI onset plotted in 3-week intervals. (**D**) Median numbers of administered EVP cycles in the overall population, responders (CR + PR), and CR population are shown in bar plots. Small circles represent individual cycle counts, and error bars indicate the interquartile range. (**E**) EV dose modifications were managed according to stepwise 0.25 mg/kg dose reductions. Events involving dose reductions per patient were analyzed using Kaplan–Meier methods. The plots report dose reductions only and do not capture dose interruptions or discontinuations. (**F**) Incidence rates of EV and pembrolizumab dose modifications or interruptions are shown in bar plots. 1L: first-line; AESI: Adverse Event of Special Interest; CR: complete response; EVP: enfortumab vedotin plus pembrolizumab; ILD: interstitial lung disease; la/mUC: locally advanced or metastatic urothelial carcinoma; PR: partial response.

In the safety analysis set of 40 patients, all-grade TRAEs were reported in 36 patients (90%), with grade 1–2 events occurring in 30 patients (75%) and grade 3–4 toxicities in 6 patients (15%). No grade 5 TRAEs were observed in this cohort. Details of EV-related and pembrolizumab-related adverse events are presented in Table S4 and Table S5. The most common grade 1–2 TRAEs were skin toxicity (55%), anorexia (43%), anemia (38%), and dysgeusia (33%) (Figure 4A). Grade 3–4 TRAEs included skin toxicity (7.5%), anorexia (5.0%), anemia (5.0%), gastrointestinal disorders (2.5%), renal dysfunction (2.5%), and ILD (2.5%). Most AESIs occurred within 3 months, whereas the onset or grade increase of peripheral neuropathy was more frequent after 3 months (Figure 4B). The incidence of the first AESI onset demonstrated a temporal pattern characterized by either an acute peak (e.g., skin toxicity) or a delayed peak (e.g., peripheral neuropathy) (Figure 4C). The median number of administered EV cycles was 4 (range, 1–13), 5 (2–13), and 10 (4–13) in the overall population, responders (CR + PR), and CR population, respectively (Figure 4D). A similar trend was observed for the number of pembrolizumab cycles. Dose modifications and interruptions of EV were common, with most patients experiencing at least one dose reduction within the first 2 months, whereas dose modifications after 4 months were uncommon (Figure 4E). However, pembrolizumab dose interruptions due to TRAEs occurred less frequently than those of EV (Figure 4F).

### Subgroup analysis of ORR and safety profile according to patient host conditions in 1L EVP therapy

**Figure 5:**
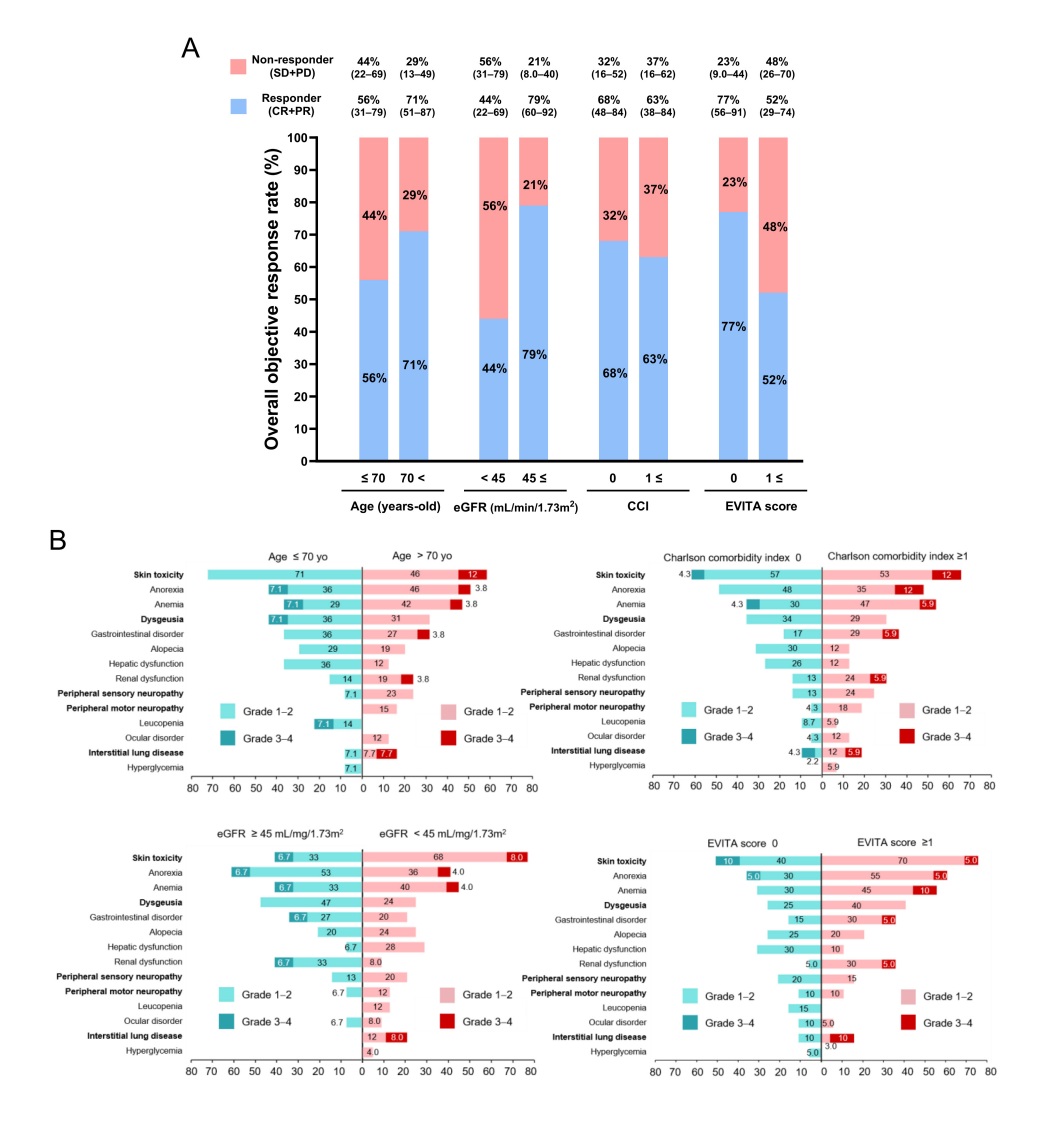
**Subgroup analysis of objective response rate and safety profile according to patient host conditions.** (**A**) Overall objective response rates to 1L EVP compared between subgroups: ≤ 70 years versus > 70 years, eGFR ≤ 45 versus > 45 mL/min/1.73 m^2^, CCI = 0 versus > 1, and EVITA score = 0 versus > 1. Responder (CR + PR) and non-responder (SD + PD) rates are presented with 95% CIs. (**B**) Subgroup analysis of safety profile in patients treated with 1L EVP for la/mUC. Treatment-related adverse events were evaluated separately based on the discretion of physicians or investigators and compared according to patient host conditions*.* 1L: first-line; CCI: Charlson Comorbidity Index; CR: complete response; eGFR: estimated glomerular filtration rate; EVITA: Enfortumab Vedotin Ineligible Criteria; EVP: enfortumab vedotin plus pembrolizumab; la/mUC: locally advanced or metastatic urothelial carcinoma; PD: progressive disease; PR: partial response; SD: stable disease.

Additionally, to investigate whether certain host-related conditions rather than tumor status were associated with the response to 1L EVP, we performed a subgroup analysis comparing responders and non-responders in 55 patients treated with 1L EVP. Among the evaluated parameters, age > 70 years, adequate renal function (eGFR ≥ 45 mL/min/1.73 m^2^), and an EVITA score of 0 were associated with a higher ORR compared with their respective counterparts (Figure 5A). In the safety analysis set of 40 patients, age (cutoff: 70 years) and CCI (0 or ≥ 1) did not significantly affect the incidence of TRAEs, whereas eGFR < 45 mL/min/1.73 m^2^ and EVITA score ≥ 1 were associated with a higher incidence of skin toxicity (Figure 5B).

## Discussion

This Japanese real-world analysis confirmed the favorable oncologic efficacy and acceptable tolerability of 1L EVP in clinical practice. Recently updated results of the EV-302 trial showed that 1L EVP achieved an overall ORR of 67.5% (95% CI: 62.9–71.9), including a CR rate of 30.4%, a PR rate of 37.1%, and a DCR of 86.5%. In our real-world cohort, a comparable ORR (57%) was observed; however, a slightly lower DCR (77%) and a markedly lower CR rate (8.6%) were noted, while the PR rate was higher (49%). Several retrospective real-world analyses of 1L EVP have reported a range of ORRs: 54% in the UNITE study (*n* = 171) [27], 60.7% in the GUARDIANS study (*n* = 164) [28], 56.6% in Austrian real-world data (*n* = 138) [29], 75% in the Mayo Clinic real-world data (*n* = 120) [30], and 63% in the Memorial Sloan Kettering Cancer Center real-world data (*n* = 232) [31]. In our cohort, the treatment response to 1L EVP was clearly higher than that of conventional CT, including GC, DD-MVAC, and GCarbo. Notably, the treatment response was not affected by the presence of visceral or liver mets.

The Canadian Bladder Cancer Information System (CBCIS) prospectively maintained database of patients who received systemic therapy for la/mUC across 15 academic institutions in Canada. A study from the CBCIS demonstrated that CR was observed in four (1.1%), PR or SD in 121 (32.7%), and PD in 99 (26.8%) in 370 patients receiving Pl-CT [32]. The CR rates were lower in the CBCIS database than our database (1.1% vs. 6.3%). Low response rate has been a significant unmet need in this disease subset for a long time. Kardoust Parizi et al. [13] conducted a systematic review and network meta-analysis including 9,082 patients with la/mUC to evaluate the ORR, OS, and PFS across different metastatic sites. The combination of durvalumab and tremelimumab as 1L systemic therapy was associated with improved OS compared with CT in patients with visceral mets (hazard ratio [HR]: 0.81) [13]. Pembrolizumab as second-line systemic therapy was also associated with better OS than chemotherapy in patients with visceral mets (HR: 0.75). Atezolizumab as 2L systemic therapy demonstrated improved OS compared with chemotherapy in patients with liver mets (HR: 0.51) and LN mets (HR: 0.59). These findings suggest that ICI therapy should be considered for patients with visceral, hepatic, or nodal metastases in both the 1L and 2L settings. Currently, multiple therapeutic regimens are available, including ADC plus ICI combinations, GC plus ICI, Pl-CT, and ICI monotherapy. Further investigations into organotropism-related differential responses are needed to optimize treatment selection. Notably, the organ-specific ORR was 100% in liver metastatic lesions in our cohort. We previously reported that the liver mets-specific response to late-line EV monotherapy was approximately 50% [33], whereas another study reported a response rate of 90.9% [34]. That article reviewed phase 2 and 3 clinical trials of ICIs, ADCs, and their combinations to highlight safe and effective strategies for individualized treatment selection. Increasing clinical and preclinical evidence indicates that pembrolizumab activates the immune system and EV exerts a direct cytotoxic effect, leading to a synergistic antitumor effect when combined [35].

Another issue that should be discussed on 1L EVP therapy is its cost-effectiveness. Wu et al. [36] calculated costs, quality-adjusted life years (QALYs), and incremental cost-effectiveness ratios (ICERs) over a lifetime horizon using EV-302 data and a parametric survival-based state-transition Markov model [36]. The economic model analysis concluded 1L EVP is not cost-effective compared with 1L CT in the UK. A similar analysis across the UK, US, and China demonstrated the current pricing makes it economically unfeasible in most settings, while EVP offered significant survival benefits for patients with la/mUC as 1L therapy. [37, 38] Because the real-world budget impact of high-cost treatments varies among countries, healthcare and economic perspectives should be evaluated in each country.

Roberson et al. [39] from the Mayo Clinic reported the largest contemporary cohort, comprising 28 patients with stage III–IV advanced UC who received EV and/or ICI systemic therapy followed by consolidative surgery [39]. Their findings suggested that consolidative surgery following EV and/or ICI treatment in carefully selected patients with advanced UC is safe, feasible, and associated with favorable rates of pathologic downstaging and promising early oncologic outcomes. Organ-specific responses and organotropism-related differential responses may serve as useful indicators for identifying patients likely to benefit from consolidative surgery. Only two (3.6%) out of 55 patients in our cohort underwent removal of the primary site as a consolidative surgery. It was not practical to evaluate oncologic outcomes and conclude the benefit of this consolidative surgery.

With respect to TRAEs, no new safety signals were identified in our analysis. The most frequently observed TRAE was skin toxicity (grade 1–2 in 55% and grade 3–4 in 7.5%), typically occurring within the initial 3 weeks after the initiation of EVP therapy. Dose reductions of EV were often required due to skin toxicity during the early treatment phase, and no further dose reductions were observed after 4 months. A systematic review and meta-analysis of EV-treated patients reported that 49.7% experienced skin toxicities, which is comparable to our findings [40]. Another TRAE warranting discussion is peripheral neuropathy, which generally occurs around 3 months after the initiation of EV therapy and can negatively impact patients’ quality of life and daily functioning. The reported incidence rates of peripheral neuropathy were 51.8% in the EV-302 trial, 42.3% in the GUARDIANS real-world cohort, 38.9% in the Austrian real-world cohort, and 24% in our real-world cohort [5, 6, 28, 29, 33]. Generally, retrospective evaluations of adverse events tend to underestimate their true incidence and severity.

The present study has some limitations that warrant acknowledgement. First, its retrospective design implies a potential for selection bias, and the decision criteria for systemic therapy, timing of dose reduction or interruption, and intervals of radiographic evaluation depended on institutional protocols and physician discretion. Second, the cohort was derived from multiple institutions, which may have introduced variability in surgical expertise, clinical judgment, and radiographic interpretation. Third, the follow-up period was relatively short, limiting the assessment of OS. Considering the potential long-term benefits of 1L EVP in this population, extended follow-up is warranted to obtain more definitive survival estimates. Fourth, the statistical power may have been limited by the small sample size. Fifth, cutaneous adverse events were not routinely evaluated by skin biopsy in our institutes. Histopathologic assessment may help identify suspected drug and supportive management.

In conclusion, this study provides real-world evidence on short-term oncologic outcomes and the safety profile of patients treated with 1L EVP. We focused on comparing the ORR and DOR between 1L EVP and conventional chemotherapy, as well as on the organotropism-related differential response. Despite the relatively short follow-up duration, our findings reflect a substantial shift in the treatment landscape for la/mUC, as this combination therapy has achieved the highest ORR reported to date for this disease subset.

## Abbreviations

1L: first-line
ADC: antibody-drug conjugate
AESI: Adverse Event of Special Interest
BSC: best supportive care
CALLY: C-reactive protein–albumin–lymphocyte
CBCIS: Canadian Bladder Cancer Information System
CCI: Charlson Comorbidity Index
CR: complete response
CRP: C-reactive protein
CT: chemotherapy
DCR: disease control rate
DD-MVAC: dose-dense methotrexate, vinblastine, doxorubicin, and cisplatin
DOR: duration of response
ECOG-PS: Eastern Cooperative Oncology Group Performance Status
eGFR: estimated glomerular filtration rate
EVITA: Enfortumab Vedotin Ineligible Criteria
EVP: enfortumab vedotin plus pembrolizumab
GC: gemcitabine plus cisplatin
GCarbo: gemcitabine plus carboplatin
HR: hazard ratio
ICI: immune checkpoint inhibitors
ILD: interstitial lung disease
la/mUC: locally advanced or metastatic urothelial carcinoma
LN: lymph node
mets: metastasis
MLR: monocyte-to-lymphocyte ratio
NLR: neutrophil-to-lymphocyte ratio
ORRs: objective response rates
OS: overall survival
PD: progressive disease
PFS: progression-free survival
Pl-CT: platinum-based chemotherapy
PLR: platelet-to-lymphocyte ratio
PMDA: Pharmaceuticals and Medical Devices Agency
PR: partial response
RECIST: Response Evaluation Criteria in Solid Tumors
SD: stable disease
SII: systemic immune-inflammation index
SIRI: systemic inflammation response index
TRAE: treatment-related adverse event
UC: urothelial carcinoma

## Supplementary materials

The supplementary material for this article is available at: https://www.explorationpub.com/uploads/Article/file/1002379_sup_1.pdf.

## Declarations

### Availability of data and materials

The data that support the findings of this study are available from the corresponding author upon reasonable request.

### Copyright

© The Author(s) 2026.
