# Supplementary material for: Oncologic outcome and safety profile of first-line enfortumab vedotin plus pembrolizumab vs. conventional chemotherapy in advanced urothelial cancer
Source: Explor Target Antitumor Ther. 2026 Jul 6;7:1002379. doi: 10.37349/etat.2026.1002379 (PMC13337572; doi:10.37349/etat.2026.1002379)
Supplement: Supplementary file 1 [file 1002379_sup_1.pdf]

# Supplementary files

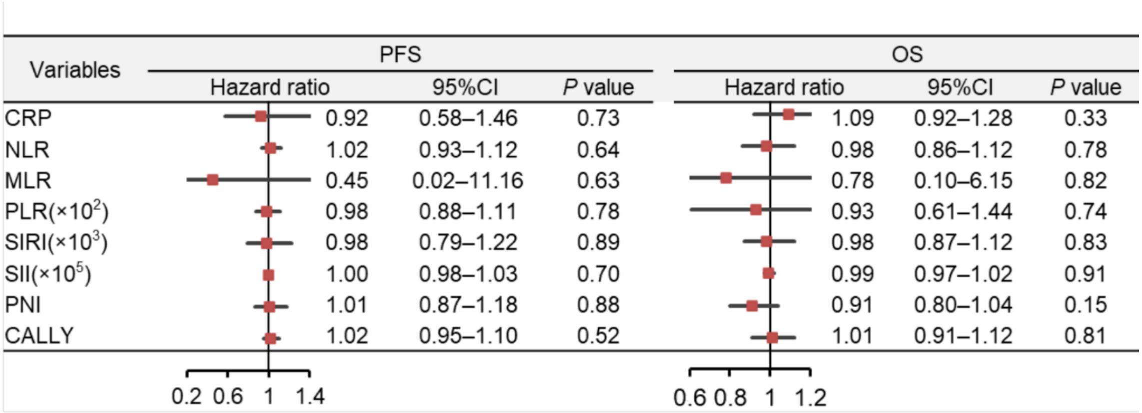


**Figure S1. Association of progression-free survival (PFS) and overall survival (OS) withblood-basedinflammationandnutritionmarkersinla/mUCpatientstreatedwith 1L EVP.** 1L: first-line; CALLY: C-reactive protein–albumin–lymphocyte; CRP: C-reactive protein; EVP: enfortumab vedotin plus pembrolizumab; la/mUC: locally advanced or metastatic urothelial carcinoma; MLR: monocyte-to-lymphocyte ratio; NLR: neutrophil-to-lymphocyte ratio; PLR: platelet-to-lymphocyte ratio; SII: systemic immune-inflammation index; SIRI: systemic inflammation response index.

**Table S1. Details of patients diagnosed with la/mUC between October 2024 and December 2025 who did not receive 1L EVP therapy.**

| ID | Age | Sex | Clinical T category (the 8th edition) | Unresectable or metastatic lesions | Radical surgery | ECOG-PS | eGFR mL/min /1.73m$^2$ | Diabetes mellitus | Number of EVITA criteria | Selected 1L therapy | Best objective response to 1L therapy RECIST ver1.1 | Reason(s) not to select EVP |
|---|---|---|---|---|---|---|---|---|---|---|---|---|
| 1 | 80 | M | 3 | Lung | No | 2 | 46 | No | 1 | BSC | NE | Rapid progression during treatment of other comorbidity (common bile duct stones) |

| | | | | | | | | | | | | |
|---|---|---|---|---|---|---|---|---|---|---|---|---|
| 2 | 79 | M | 4 | Primary tumor<br>Local dissemination<br>Lymph node | No | 0 | 40 | No | 1 | DD-MVACarbo | PD | i) Previous use of pembrolizumab for advanced lung cancer<br>ii) Pembrolizumab caused interstitial lung disease at that time<br>iii) Urothelial carcinoma diagnosed after pembrolizumab treatment (highly suspected primary |

| | | | | | | | | | | | | |
|---|---|---|---|---|---|---|---|---|---|---|---|---|
| | | | | | | | | | | | | resistance to pembrolizumab) |
| 3 | 79 | M | 3 | Local recurrence<br>Lymph node | RNU | 0 | 33 | Yes (HbA1c, 7.6%) | 1 | GEM + Carbo | PR | The attending physician had no experience with EVP use |
| 4 | 85 | F | 1 | Lymph node<br>Lung | No | 1 | 27 | No | 1 | GEM + Carbo | PD | Because of high age |
| 5 | 77 | M | 2 | Local recurrence<br>Lymph node | RC | 1 | 41 | Yes (Glucose level, | 1 | GC | PR | The attending physician had no experience with EVP use |

| | | | | | | | | | | | | |
|---|---|---|---|---|---|---|---|---|---|---|---|---|
| | | | | | | | | 182mg/dL ) | | | | |
| 6 | 86 | M | 3 | Lymph node | No | 0 | 52 | No | 0 | GEM + Carbo | NE | The patients refused EVP after sufficient shared decision-making |
| 7 | 76 | M | 4 | Lymph node | No | 0 | 59 | Yes (HbA1c 9.0%) | 1 | DD-MVACarbo | NE | Uncontrolled diabetes mellitus |
| 8 | 77 | F | 3 | Local recurrence<br>Lymph node | RNU | 1 | 27 | No | 1 | GEM + Carbo | SD | i) Because of high age<br>ii) Urothelial carcinoma diagnosed during |

| | | | | | | | | | | | | |
|---|---|---|---|---|---|---|---|---|---|---|---|---|
| | | | | | | | | | | | | adjuvant nivolumab treatment (highly suspected primary resistance to PD-1 inhibitors) |
| 9 | 77 | M | 4 | Lung | RNU | 1 | 38 | No | 1 | DD-MVACarbo | PR | The attending physician had no experience with EVP use |
| 10 | 84 | M | 3 | Primary tumor | No | 1 | 50 | Yes (HbA1c 6.4%) | 1 | GEM + Carbo | PR | Because of high age |

1L: first-line; ACTH: adrenocorticotropic hormone; BSC: best supportive care; DD-MVAC: dose-dense methotrexate, vinblastine, adriamycin, and cisplatin combination chemotherapy; ECOG-PS: Eastern Cooperative Oncology Group Performance Status; eGFR: estimated glomerular

filtration rate; EVITA: Enfortumab Vedotin Ineligible Criteria; EVP: enfortumab vedotin plus pembrolizumab combination therapy; FT3: free triiodothyronine; FT4: free thyroxine; GC: gemcitabine plus cisplatin combination chemotherapy; IQR: interquartile range; la/mUC: locally advanced or metastatic urothelial carcinoma; NA: not available; RECIST: Response Evaluation Criteria in Solid Tumors; SLD: sum of longest diameter; TSH: thyroid-stimulating hormone; UTUC: upper urinary tract urothelial carcinoma.

**Table S2. Baseline characteristics of patients with la/mUC: comparison among selected 1L treatment.**

| **Variables** | | **Total** | **EVP** | **GC** | **DD-MVAC** | **GCarbo** | **Other** | **BSC** | ***P* value** |
|---|---|---|---|---|---|---|---|---|---|
| Total | | 642 (100%) | 55 (8.6%) | 281 (43.8%) | 13 (2.0%) | 178 (27.7%) | 50 (7.8%) | 65 (10.1%) | - |
| Age, years-old | Median (IQR) | 75 (68–79) | 74 (66–78) | 73 (66–78) | 76 (73–78) | 75 (71–79) | 75 (71–79) | 80 (74–87) | < 0.01 |
| Sex | Male | 459 (71%) | 40 (73%) | 206 (73%) | 13 (100%) | 125 (70.2%) | 40 (80%) | 35 (53.8%) | < 0.01 |
| | Female | 183 (29%) | 15 (27%) | 75 (27%) | 0 | 53 (29.8%) | 10 (20%) | 30 (46.2%) | |
| Smoking history | Never | 213 (33%) | 17 (31%) | 83 (29%) | 4 (31%) | 68 (38%) | 17 (34%) | 24 (37%) | 0.11 |
| | Former | 182 (28%) | 20 (36%) | 73 (26%) | 6 (46%) | 50 (28%) | 18 (36%) | 15 (23%) | |

| | | | | | | | | | |
|---|---|---|---|---|---|---|---|---|---|
| | Current | 87 (14%) | 10 (18%) | 42 (15%) | 3 (23%) | 20 (11%) | 6 (12%) | 6 (9.2%) | |
| | Unknown | 160 (25%) | 8 (15%) | 83 (30%) | 0 | 40 (22%) | 9 (18%) | 20 (30%) | |
| Charlson Comorbidity Score-Category | None (score 0) | 394 (61%) | 29 (53%) | 189 (67%) | 8 (62%) | 119 (67%) | 24 (48%) | 25 (38%) | < 0.01 |
| | Mild (score 1 or 2) | 188 (29%) | 22 (40%) | 66 (23%) | 3 (23%) | 50 (28%) | 22 (44%) | 25 (38%) | |
| | Moderate (score 3 or 4) | 36 (5.6%) | 4 (7.3%) | 11 (3.9%) | 2 (15%) | 8 (4.5%) | 2 (4.0%) | 9 (14%) | |
| | Severe (score 5 or more) | 24 (3.7%) | 0 | 15 (5.3%) | 0 | 1 (0.6%) | 2 (4.0%) | 6 (9.2%) | |

| | | | | | | | | | |
|---|---|---|---|---|---|---|---|---|---|
| Diabetes mellitus | No | 560 (87%) | 48 (87%) | 246 (88%) | 10 (77%) | 157 (88%) | 46 (92%) | 53 (82%) | 0.51 |
| | Yes | 82 (13%) | 7 (13%) | 35 (12%) | 3 (23%) | 21 (12%) | 4 (8.0%) | 12 (18%) | |
| The use of steroids at baseline | No | 633 (99%) | 55 (100%) | 275 (98%) | 13 (100%) | 177 (99%) | 49 (98%) | 64 (98%) | 0.69 |
| | Yes | 9 (1.4%) | 0 | 6 (2.1%) | 0 | 1 (0.6%) | 1 (2.0%) | 1 (1.5%) | |
| Autoimmune disease | No | 626 (98%) | 54 (98%) | 273 (97%) | 13 (100%) | 175 (98%) | 48 (96%) | 63 (97%) | 0.90 |
| | Yes | 16 (2.5%) | 1 (1.8%) | 8 (2.8%) | 0 | 3 (1.7%) | 2 (4.0%) | 2 (3.1%) | |
| Primary disease | Bladder | 327 (51%) | 29 (53%) | 164 (58%) | 5 (38%) | 70 (39%) | 25 (50%) | 34 (52%) | 0.05 |
| | Renal pelvis | 171 (27%) | 15 (27%) | 70 (25%) | 5 (38%) | 53 (30%) | 13 (26%) | 15 (23%) | |
| | Ureter | 138 (21%) | 11 (20%) | 43 (15%) | 3 (23%) | 53 (30%) | 12 (24%) | 16 (25%) | |
| | Undefined | 6 (0.9%) | 0 | 4 (1.4%) | 0 | 2 (1.1%) | 0 | 0 | |
| Concomitant bladder cancer and UTUC | No | 633 (99%) | 54 (98%) | 281 (100%) | 13 (100%) | 174 (98%) | 48 (96%) | 63 (97%) | 0.11 |

| | | | | | | | | | |
|---|---|---|---|---|---|---|---|---|---|
| | Yes | 9 (1.4%) | 1 (1.8%) | 0 | 0 | 4 (2.2%) | 2 (4.0%) | 2 (3.1%) | |
| Radical surgery | None | 191 (30%) | 30 (55%) | 82 (29%) | 9 (69%) | 43 (24%) | 9 (18%) | 18 (28%) | <0.01 |
| | Cystectomy | 227 (35%) | 16 (29%) | 127 (45%) | 2 (15%) | 38 (21%) | 18 (36%) | 26 (40%) | |
| | Nephroureterectomy | 197 (31%) | 8 (15%) | 69 (25%) | 2 (15%) | 85 (48%) | 18 (36%) | 15 (23%) | |
| | Both | 27 (4.2%) | 1 (1.8%) | 3 (1.1%) | 0 | 12 (6.7%) | 5 (10%) | 6 (9.2%) | |
| Unresectable or metastatic lesions | Local lesion | 293 (46%) | 31 (56%) | 139 (49%) | 8 (62%) | 69 (39%) | 20 (40%) | 26 (40%) | 0.07 |
| | Lymph nodes | 402 (63%) | 40 (73%) | 172 (61%) | 10 (80%) | 116 (65%) | 32 (64%) | 32 (49%) | 0.10 |
| | Lung | 184 (29%) | 17 (31%) | 83 (30%) | 1 (7.7%) | 53 (30%) | 10 (20%) | 20 (31%) | 0.40 |
| | Liver | 67 (10%) | 13 (24%) | 23 (8.2%) | 1 (7.7%) | 17 (9.6%) | 4 (8.0%) | 9 (14%) | 0.13 |
| | Bone | 78 (12%) | 9 (16%) | 37 (13%) | 0 | 18 (10%) | 5 (10%) | 9 (14%) | 0.54 |
| | Peritoneum | 28 (4.4%) | 2 (3.6%) | 10 (36%) | 1 (7.7%) | 7 (3.9%) | 2 (4.0%) | 6 (9.2%) | 0.46 |

| | | | | | | | | | |
|---|---|---|---|---|---|---|---|---|---|
| | Retroperitoneum | 12 (1.9%) | 7 (13%) | 3 (1.1%) | 0 | 0 | 2 (4.0%) | 0 | < 0.01 |
| | Adrenal grand | 11 (1.7%) | 1 (1.8%) | 4 (1.4%) | 0 | 4 (2.3%) | 0 | 2 (3.1%) | 0.81 |
| SLD at baseline (mm), RECIST v1.1 | Median (IQR) | 38 (20–60) | 49 (34–81) | 40 (19–63) | 47 (30–63) | 33 (18–51) | 28 (17–39) | 37 (27–42) | 0.01 |
| EVITA criteria | HbA1c ≥ 8% | 8 (1.3%) | 1 (1.8%) | 5 (1.8%) | 1 (7.7%) | 1 (0.6%) | 0 | 0 | 0.12 |
| | Grade ≥ 2 sensory or motor neuropathy | 18 (2.8%) | 0 | 5 (1.8%) | 0 | 5 (2.8%) | 0 | 8 (12%) | < 0.01 |

| | | | | | | | | | |
|---|---|---|---|---|---|---|---|---|---|
| | Any corneal or retinal abnormality | 11 (1.7%) | 1 (1.8%) | 2 (0.7%) | 0 | 2 (1.1%) | 0 | 5 (7.7%) | < 0.01 |
| | eGFR < 45 mL/min/1.73 $m^2$ | 226 (35%) | 22 (40%) | 51 (18%) | 6 (46%) | 115 (65%) | 31 (62%) | 1 (1.5%) | < 0.01 |
| | ECOG-PS ≥ 2 | 48 (7.5%) | 6 (11%) | 22 (7.8%) | 0 | 9 (5.1%) | 4 (8.0%) | 12 (18%) | 0.01 |
| Number of EVITA criteria | 0 | 368 (57%) | 29 (53%) | 209 (74%) | 6 (46%) | 59 (33%) | 18 (36%) | 47 (72%) | < 0.01 |
| | 1 | 241 (38%) | 22 (40%) | 62 (22%) | 7 (54%) | 110 (62%) | 29 (58%) | 11 (17%) | |
| | 2 | 26 (4.0%) | 4 (7.3%) | 8 (2.9%) | 0 | 5 (2.8%) | 3 (6.0%) | 6 (9.2%) | |
| | 3 | 7 (1.1%) | 0 | 2 (0.7%) | 0 | 4 (2.3%) | 0 | 1 (1.5%) | |
| Laboratory data, median (IQR) | Neutrophil, × $10^3/\mu L$ | 4.1 (3.1–5.6) | 4.7 (3.5–5.9) | 4.2 (3.2–5.7) | 5.1 (4.4–6.9) | 3.9 (3.0–5.1) | 3.5 (2.5–5.1) | 5.3 (4.3–5.6) | 0.04 |

| | | | | | | | | |
|---|---|---|---|---|---|---|---|---|
| Alb, g/dL | 3.8 (3.4–4.1) | 3.9 (3.7–4.1) | 3.7 (3.2–4.0) | 3.8 (3.6–4.0) | 3.9 (3.5–4.1) | 4.0 (3.5–4.2) | 3.5 (3.2–3.7) | 0.03 |
| eGFR, mL/min/1.73m$^2$ | 49.0 (39.5–61.2) | 48.5 (39.3–57.9) | 56.0 (47.8–65.9) | 53.2 (40.7–61.4) | 39.9 (33.8–46.3) | 39.6 (27.4–53.0) | 52.3 (47.3–75.3) | < 0.01 |
| ACTH, pg/mL | 26.4 (18.6–37.0) | 27.6 (18.8–37.0) | NA | NA | 19.9 (19.0–20.8) | NA | NA | 0.62 |
| Cortisol, μg/dL | 12.3 (8.5–17.01) | 12.4 (8.7–17.1) | NA | NA | 8.6 (8.2–9.1) | NA | NA | 0.44 |
| FT3, ng/dL | 2.50 (2.12–2.72) | 2.43 (2.03–2.75) | NA | NA | 2.6 (2.5–2.7) | NA | NA | 0.68 |

| | | | | | | | | | |
|---|---|---|---|---|---|---|---|---|---|
| | FT4, ng/dL | 1.11 (0.99–1.28) | 1.12 (0.99–1.29) | NA | NA | 0.9 (0.9–1.0) | NA | NA | 0.28 |
| | TSH, µIU/mL | 1.94 (1.34–3.06) | 1.95 (1.32–3.05) | NA | NA | 3.8 (2.8–4.7) | NA | NA | 0.45 |

1L, first-line; ACTH: adrenocorticotropic hormone; BSC: best supportive care; DD-MVAC: dose-dense methotrexate, vinblastine, adriamycin, and cisplatin combination chemotherapy; ECOG-PS: Eastern Cooperative Oncology Group Performance Status; eGFR: estimated glomerular filtration rate; EVITA: nfortumab Vedotin Ineligible criTeriA; EVP: enfortumab vedotin plus pembrolizumab combination therapy; FT3: free triiodothyronine; FT4: free thyroxine; GC: gemcitabine plus cisplatin combination chemotherapy; GCarbo: gemcitabine plus carboplatin combination chemotherapy; IQR: interquartile range; la/mUC: locally advanced or metastatic urothelial carcinoma; NA: not available; RECIST: Response Evaluation Criteria in Solid Tumors; SLD: sum of longest diameter; TSH: thyroid stimulating hormone; UTUC: upper urinary tract urothelial carcinoma.

**Table S3. Baseline characteristics of patients with bladder cancer and UTUC.**

| **Variables** | | **Total** | **Bladder** | **UTUC** | ***P* value** |
|---|---|---|---|---|---|
| Total | | 55 | 29 | 26 | 0.61 |
| Age, years-old | Median (IQR) | 74 (66–78) | 73 (66–78) | 75 (69–78) | |
| Sex | Male | 40 (73%) | 26 (90%) | 14 (54%) | 0.005 |
| | Female | 15 (27%) | 3 (10%) | 12 (46%) | |
| Smoking history | Never | 17 (31%) | 8 (28%) | 9 (35%) | 0.88 |
| | Former | 20 (36%) | 10 (35%) | 10 (39%) | |
| | Current | 10 (18%) | 6 (21%) | 4 (15%) | |
| | Unknown | 8 (15%) | 5 (17%) | 3 (12%) | |
| Charlson Comorbidity Score-Category | None (score 0) | 33 (60%) | 16 (55%) | 17 (65%) | 0.68 |
| | Mild (score 1 or 2) | 16 (29%) | 9 (31%) | 7 (27%) | |

| | | | | | |
|---|---|---|---|---|---|
| | Moderate (score 3 or 4) | 4 (7.3%) | 2 (6.9%) | 2 (7.7%) | |
| | Severe (score 5 or more) | 2 (3.6%) | 2 (6.9%) | 0 (0.0%) | |
| Diabetes mellitus | No | 48 (87%) | 23 (79%) | 25 (96%) | 0.11 |
| | Yes | 7 (13%) | 6 (21%) | 1 (3.8%) | |
| The use of steroids at baseline | No | 55 (100%) | 29 (100%) | 26 (100%) | NA |
| | Yes | 0 | 0 | 0 | |
| Autoimmune disease | No | 54 (98%) | 28 (97%) | 26 (100%) | 1 |
| | Yes | 1 (1.8%) | 1 (3.4%) | 0 | |
| Radical surgery | None | 30 (55%) | 13 (45%) | 17 (65%) | <0.001 |
| | Cystectomy | 16 (29%) | 16 (55%) | 0 (0%) | |
| | Nephroureterectomy | 8 (15%) | 0 | 8 (31%) | |
| | Both | 1 (1.8%) | 0 | 1 (3.8%) | |

| | | | | | |
|---|---|---|---|---|---|
| Metastatic lesions | Local lesion | 31 (56%) | 14 (48%) | 17 (65%) | 0.28 |
| | Lymph nodes | 40 (73%) | 22 (76%) | 18 (69%) | 0.76 |
| | Lung | 17 (31%) | 10 (35%) | 7 (27%) | 0.57 |
| | Liver | 11 (20%) | 6 (21%) | 5 (19%) | 1.00 |
| | Bone | 9 (16%) | 5 (17%) | 4 (15%) | 1.00 |
| | Peritoneum | 2 (3.6%) | 1 (3.4%) | 1 (3.8%) | 1.00 |
| | Retroperitoneum | 7 (13%) | 1 (3.4%) | 6 (23%) | 0.04 |
| | Adrenal grand | 1 (1.8%) | 1 (3.4%) | 0 | 1.00 |
| SLD at baseline (mm), RECIST v1.1 | Median (IQR) | 49.8 (34.2–80.5) | 47.2 (34.0–64.3) | 53.5 (40.2–93.7) | 0.34 |
| EVITA score | 0 | 29 (53%) | 16 (55%) | 13 (50%) | 0.60 |
| | 1 | 22 (40%) | 12 (41%) | 10 (39%) | |
| | 2 | 4 (7.3%) | 1 (3.4%) | 3 (12%) | |

| | | | | | |
|---|---|---|---|---|---|
| | 3 | 0 | 0 | 0 | |
| EVITA criteria | HbA1c ≥8% | 1 (3.0%) | 1 (5.9%) | 0 (0%) | 1.00 |
| | Grade ≥ 2 neuropathy | 0 | 0 | 0 | NA |
| | Any corneal or retinal abnormality | 1 (1.8%) | 1 (3.4%) | 0 (0%) | 1.00 |
| | eGFR < 45 mL/min/1.73 $m^2$ | 22 (41%) | 9 (32%) | 13 (50%) | 0.27 |
| | ECOG-PS ≥ 2 | 6 (11%) | 3 (10%) | 3 (12%) | 1.00 |
| The number of treatment cycles | Median (IQR) | 3 (2–5) | 4 (2–7) | 4 (2–8) | 0.86 |
| Laboratory data, median (IQR) | Neutrophil, $\times 10^3/\mu L$ | 4.7 (3.5–5.9) | 5.1 (3.8–5.9) | 3.8 (3.3–5.8) | 0.16 |
| | Alb, g/dL | 3.9 (3.7–4.1) | 3.9 (3.7–4.1) | 3.9 (3.7–4.0) | 0.67 |
| | eGFR, mL/min/1.73$m^2$ | 48.5 (39.3–57.9) | 51.1 (41.2–63.8) | 44.9 (37.5–51.8) | 0.04 |
| | ACTH, pg/mL | 27.6 (18.8–37.0) | 22.3 (15.8–34.9) | 31.4 (21.0–38.8) | 0.13 |
| | Cortisol, µg/dL | 12.4 (8.7–17.1) | 12.3 (8.6–17.2) | 12.4 (9.5–17.1) | 0.81 |

| | | | | | |
|---|---|---|---|---|---|
| | FT3, ng/dL | 2.43 (2.03–2.75) | 2.66 (2.25–2.87) | 2.34 (1.88–2.62) | 0.04 |
| | FT4, ng/dL | 1.12 (0.99–1.29) | 1.03 (0.99–1.23) | 1.17 (1.07–1.30) | 0.17 |
| | TSH, μIU/mL | 1.95 (1.32–3.05) | 1.83 (1.19–2.83) | 2.36 (1.56–3.69) | 0.21 |

1L, first-line; ACTH: adrenocorticotropic hormone; BSC: best supportive care; DD-MVAC: dose-dense methotrexate, vinblastine, adriamycin, and cisplatin combination chemotherapy; ECOG-PS: Eastern Cooperative Oncology Group Performance Status; eGFR: estimated glomerular filtration rate; EVITA: nfortumab Vedotin Ineligible criTeriA; EVP: enfortumab vedotin plus pembrolizumab combination therapy; FT3: free triiodothyronine; FT4: free thyroxine; GC: gemcitabine plus cisplatin combination chemotherapy; GCarbo: gemcitabine plus carboplatin combination chemotherapy; IQR: interquartile range; la/mUC: locally advanced or metastatic urothelial carcinoma; NA: not available; RECIST: Response Evaluation Criteria in Solid Tumors; SLD: sum of longest diameter; TSH: thyroid stimulating hormone; UTUC: upper urinary tract urothelial carcinoma.

**Table S4. Details of EV-related adverse events in patients treated with first-line (1L) enfortumab vedotin plus pembrolizumab combination therapy (EVP).**

| **Adverse events** | CTCAE vs. 5.0 grading | Number of patients |
|---|---|---|
| *n* | - | 40 |
| Skin toxicity | 0 | 15 (38%) |
| | 1 | 13 (33%) |
| | 2 | 9 (23%) |
| | 3 | 3 (7.5%) |
| Anorexia | 0 | 21 (53%) |
| | 1 | 10 (25%) |
| | 2 | 7 (18%) |

| | | |
|---|---|---|
| | 3 | 1 (2.5%) |
| | 4 | 1 (2.5%) |
| Anemia | 0 | 23 (58%) |
| | 1 | 12 (30%) |
| | 2 | 3 (7.5%) |
| | 3 | 2 (5.0%) |
| Dysgeusia | 0 | 27 (68%) |
| | 1 | 9 (23%) |
| | 2 | 4 (10%) |
| Gastrointestinal disorder | 0 | 30 (75%) |
| | 1 | 7 (18%) |
| | 2 | 2 (5.0%) |

| | | |
|---|---|---|
| | 4 | 1 (2.5%) |
| Alopecia | 0 | 31 (78%) |
| | 1 | 6 (15%) |
| | 2 | 3 (7.5%) |
| Hepatic dysfunction | 0 | 32 (80%) |
| | 1 | 6 (15%) |
| | 2 | 2 (5.0%) |
| Renal dysfunction | 0 | 32 (80%) |
| | 1 | 5 (13%) |
| | 2 | 2 (5.0%) |
| | 5 | 1 (2.5%) |
| Peripheral sensory neuropathy | 0 | 33 (83%) |

| | | |
|---|---|---|
| | 1 | 5 (13%) |
| | 2 | 2 (5.0%) |
| Peripheral motor neuropathy | 0 | 36 (90%) |
| | 1 | 2 (5.0%) |
| | 2 | 2 (5.0%) |
| Leukopenia | 0 | 37 (93%) |
| | 1 | 2 (5.0%) |
| | 2 | 1 (2.5%) |
| Ocular disorder | 0 | 37 (93%) |
| | 1 | 3 (7.5) |
| Respiratory dysfunction | 0 | 37 (93%) |
| | 1 | 1 (2.5%) |

| | | |
|---|---|---|
| | 2 | 1 (2.5%) |
| | 4 | 1 (2.5%) |
| Hyperglycemia | 0 | 39 (98%) |
| | 2 | 1 (2.5%) |

**Table S5. Details of pembrolizumab-related adverse events in patients treated with first-line (1L) enfortumab vedotin plus pembrolizumab combination therapy (EVP).**

| **Adverse events** | **CTCAE ver 5.0 grading** | **Number of patients** |
|---|---|---|
| *n* | - | 40 |
| Skin toxicity | 0 | 30 (75%) |
| | 1 | 6 (15%) |
| | 2 | 3 (7.5%) |
| | 3 | 1 (2.5%) |
| Interstitial lung disease | 0 | 36 (90%) |
| | 1 | 1 (2.5%) |
| | 2 | 1 (2.5%) |

| | | |
|---|---|---|
| | 4 | 2 (5.0%) |
| Thyroid dysfunction | 0 | 37 (93%) |
| | 1 | 2 (5.0%) |
| | 2 | 1 (2.5%) |
| Renal dysfunction | 0 | 38 (95%) |
| | 1 | 1 (2.5%) |
| | 5 | 5 (13%) |
| Hepatic dysfunction | 0 | 38 (95%) |
| | 1 | 1 (2.5%) |
| | 2 | 1 (2.5%) |
